# The upper limit of the in-plane spin splitting of Gaussian beam reflected from a glass-air interface

**DOI:** 10.1038/s41598-017-01323-0

**Published:** 2017-04-25

**Authors:** Wenguo Zhu, Jianhui Yu, Heyuan Guan, Huihui Lu, Jieyuan Tang, Jun Zhang, Yunhan Luo, Zhe Chen

**Affiliations:** 1 Guangdong Provincial Key Laboratory of Optical Fiber Sensing and Communications, Jinan University, Guangzhou, 510632 China; 2 Key Laboratory of Optoelectronic Information and Sensing Technologies of Guangdong Higher Education Institutes, Jinan University, Guangzhou, 510632 China; 3 Department of Optoelectronic Engineering, Jinan University, Guangzhou, 510632 China

## Abstract

Optical spin splitting has a promising prospect in quantum information and precision metrology. Since it is typically small, many efforts have been devoted to its enhancement. However, the upper limit of optical spin splitting remains uninvestigated. Here, we investigate systematically the in-plane spin splitting of a Gaussian beam reflected from a glass-air interface and find that the spin splitting can be enhanced in three different incident angular ranges: around the Brewster angle, slightly smaller than and larger than the critical angle for total reflection. Within the first angular range, the reflected beam can undergo giant spin splitting but suffers from low energy reflectivity. In the second range, however, a large spin splitting and high energy reflectivity can be achieved simultaneously. The spin splitting becomes asymmetrical within the last angular range, and the displacement of one spin component can be up to half of incident beam waist *w*_0_/2. Of all the incident angles, the spin splitting reaches its maximum at Brewster angle. This maximum splitting increases with the refractive index of the “glass” prism, eventually approaching an upper limit of *w*_0_. These findings provide a deeper insight into the optical spin splitting phenomena and thereby facilitate the development of spin-based applications.

## Introduction

As is well known, when a light beam is reflected from or transmitted through an interface between two different media, its two opposite spin components may separate in directions parallel and perpendicular to the plane of incidence, i.e., so-called the in-plane and out-of-plane spin splitting (IPSS and OPSS)^1–3^. The IPSS is related to the angular gradients of Fresnel coefficients, while the OPSS, resulting from the spin-orbit interaction, is independent of the change of the Fresnel coefficients with the incident angle^3^. It has been demonstrated that the IPSS and OPSS can be considered as analogous but reverse effects^3^. Both of them can be rewritten as a combination of a *z*_*r*_- (propagation axis) independent term and a *z*_*r*_-dependent term, which associate with the spatial and angular spin splitting, respectively^1^. Researchers have shown more interest in the spin splitting in initial plane at *z*_*r*_ = 0, where the angular spin splitting vanishes, and the total spin splitting is equal to the spatial one^4–6^. The spatial spin splitting can serve as a useful metrological tool for characterizing the variations of nanostructure parameters, for instance, in the identification of the graphene layers^4^. However, the splitting is generally tiny and can only reach a fraction of a wavelength. Weak measurement technology or other complicated methods are therefore needed for its measurement^5–7^. To ensure its applications in quantum information and precision metrology, large spin splitting is highly desirable^8^. Many efforts have been devoted to pursuing large spatial spin splitting^9–11^. It was found that, the OPSS can be enhanced when a Gaussian beam is reflected by an air-glass interface near the Brewster angle^12, 13^. Götte and coworkers found the eigenpolarizations of the OPSS^14^. By choosing a proper incident polarization, they demonstrated a spin splitting of ten wavelengths near Brewster incidence in their experiments. Tan and Zhu took advantage of long-range surface plasmon resonance and theoretically obtained a spin separation of 7.85 *μm* with a 632.8 *nm* incident Gaussian beam^15^. However, in all of the above cases, the spin splitting values were much smaller than the incident beam waists *w*_0_. In 2015, an OPSS up to *w*_0_ was achieved when a one-dimensional (1D) Gaussian beam with *w*_0_ = 10.2 *μ*m was reflected from an air-glass interface^16^. For a two-dimensional (2D) Gaussian beam, however, the OPSS could only reach 0.4*w*_0_. It was demonstrated recently that the IPSS could also be enhanced near Brewster incidence^17^. The IPSS shows advantage over the OPSS since it can be tuned flexibly by the incident polarization state.

In this paper, we will focus our attention on the IPSS of a paraxial Gaussian beam reflected by a glass-air interface. We find that the IPSS varies with incident angle. When the incident angle is around the Brewster angle, the IPSS can be quite large. Specially at the Brewster angle, the IPSS can be approximately close to the incident beam waist *w*_0_, which is proven to be the upper limit of the IPSS. We further study the IPSSs in two other angular ranges: when the incident angle is either slightly smaller or larger than the critical angle, which is the angle of incidence for which the angle of refraction is 90°. In the former angular range, both the IPSS and the energy reflectivity increase with incident angle. Therefore, large spin splitting and high energy reflectivity can be obtained simultaneously. In the latter angular range, however, asymmetric spin splitting may occur, i.e., the displacement of one of the spin component is relatively small, while the displacement of the other component can be up to *w*_0_/2. This large displacement is extremely sensitive to the incident polarization state.

## Theory

**Figure 1:**
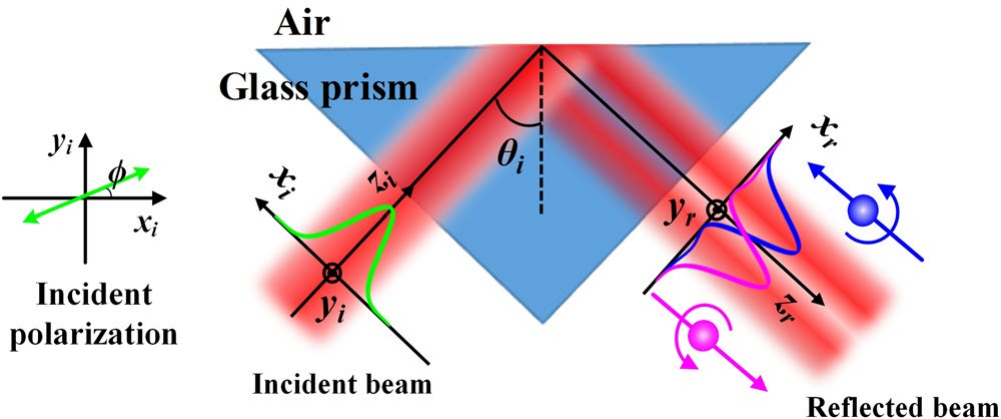
The schematic of the in-plane optical spin splitting. When a linearly polarized Gaussian beam is launched onto the glass-air interface, two opposite spin components of reflected beam shift toward opposite directions, thus split spatially.

The schematic of the generation of IPSS is shown in Fig. 1, where a Gaussian beam is launched onto the glass-air interface with an incident angle of *θ*_*i*_. The local coordinate systems attached to the incident and reflected beams are (*x*_*i*_, *y*_*i*_, *z*_*i*_) and (*x*_*r*_, *y*_*r*_, *z*_*r*_), respectively. The angular spectrum of incident beam is ${\tilde{{\bf{E}}}}_{i}=A\,\exp \,[-({k}_{ix}^{2}+{k}_{iy}^{2}){w}_{0}^{2}/4][a{\hat{{\bf{e}}}}_{ix}+b{\hat{{\bf{e}}}}_{iy}]$, where *k*_*ix*_ and *k*_*iy*_ are the transverse wavenumbers, *w*_0_ is the beam waist, **ê**_*ix*_ and **ê**_*iy*_ are the polarization unit vectors parallel and perpendicular to the incidence plane, *a* = cos*ϕ* and *b* = sin *ϕ* exp (*iδ*) with *δ* being the phase differences between *x*_*i*_ and *y*_*i*_ linear polarization components and *ϕ* determining their amplitude ratio, respectively. *A* = *w*_0_/(2*π*)^1/2^ is a constant making $\int |{\tilde{{\bf{E}}}}_{i}{|}^{2}d{k}_{ix}d{k}_{iy}=1$. The relationship between the angular spectra of the reflected and incident beams under the paraxial condition has been derived in ref. 1. According to the relationship, the angular spectrum of the reflected beam can be written as ${\tilde{{\bf{E}}}}_{r}=A\,\exp \,[-({k}_{rx}^{2}+{k}_{ry}^{2}){w}_{0}^{2}/4]$ $[a{r}_{p}{\hat{{\bf{e}}}}_{rx}+b{r}_{s}{\hat{{\bf{e}}}}_{ry}]$, where *k*_*rx*_ = −*k*_*ix*_, *k*_*ry*_ = *k*_*iy*_, and *r*_*p*_ and *r*_*s*_ are the Fresnel reflection coefficients for *p* and *s* waves, respectively. The reflection coefficients can be expanded into Taylor series. By making the first-order approximation, ${r}_{p}={r}_{p{\theta }_{i}}-{r}_{p}^{^{\prime} }{k}_{rx}/{k}_{i}$ and ${r}_{s}={r}_{s{\theta }_{i}}-{r}_{s}^{^{\prime} }{k}_{rx}/{k}_{i}$, where *r*_*p*_′ and *r*_*s*_′ are the first derivatives of reflection coefficients, *k*_*i*_ = *k0n* with *k*_*0*_ and *n* being the wavenumber in free space and the refractive index of the prism, respectively. Therefore, the electric field in real space is ref. 12

1 $${{\bf{E}}}_{r}^{\sigma }=\frac{C}{\sqrt{2}}\exp \,[-\frac{{x}_{r}^{2}+{y}_{r}^{2}}{{w}_{0}^{2}}]\{[a{r}_{p{\theta }_{i}}+\frac{ibM{y}_{r}-ia{r}_{p}^{^{\prime} }{x}_{r}}{{z}_{0}}+\frac{bN{x}_{r}{y}_{r}}{{z}_{0}^{2}}]-i\sigma [b{r}_{s{\theta }_{i}}-\frac{iaM{y}_{r}+ib{r}_{s}^{^{\prime} }{x}_{r}}{{z}_{0}}-\frac{aN{x}_{r}{y}_{r}}{{z}_{0}^{2}}]\}{\hat{{\bf{e}}}}_{r\sigma },$$

where *C* = (2/*π*)^1/2^/*w*_0_, *M* = (*r*_*p*_ + *r*_*s*_)cot*θ*_*i*_, *N* = (*r*_*p*_′ + *r*_*s*_′)cot*θ*_*i*_, *z*_0_ = *k*_*i*_ *w*_0_^2^/2, **ê**_*rσ*_ = 2^−1/2^(**ê**_*rx*_ + *iσ* **ê**_*ry*_), and *σ* = ±1 corresponding to the right and left circular polarizations (RCP and LCP), respectively. The terms in Eq. (1) containing *M* result from wave-vector spreading of the incident beam along the *y*_*i*_ axis. These terms are zero for a 1D incident Gaussian beam. To study the spin splitting, we calculate the displacements of the gravity centers of the RCP and LCP components of the reflected beam, which are defined as ${{\rm{\Delta }}}_{\sigma }=\iint {x}_{r}|{{\bf{E}}}_{r}^{\sigma }{|}^{2}d{x}_{r}d{y}_{r}/\iint |{{\bf{E}}}_{r}^{\sigma }{|}^{2}d{x}_{r}d{y}_{r}$ ^17^. By neglecting second order terms of 1/*z*_0_, we have

2 $${{\rm{\Delta }}}_{\sigma }=\frac{1}{2{k}_{i}{W}_{\sigma }}\{\text{Im}[{|a|}^{2}{r}_{p{\theta }_{i}}^{\ast }{r}_{p}^{^{\prime} }+{|b|}^{2}{r}_{s{\theta }_{i}}^{\ast }{r}_{s}^{^{\prime} }]+\sigma \,{\rm{Re}}[a{r}_{p}^{^{\prime} }{b}^{\ast }{r}_{s{\theta }_{i}}^{\ast }-{a}^{\ast }{r}_{p{\theta }_{i}}^{\ast }b{r}_{s}^{^{\prime} }]\},$$

where the energies of reflected components are

3 $$\begin{matrix}{W}_{\sigma }=\frac{1}{2}\{{|a{r}_{p{\theta }_{i}}|}^{2}+{|b{r}_{s{\theta }_{i}}|}^{2}+2\sigma \,{\rm{Im}}[{a}^{\ast }{r}_{p{\theta }_{i}}^{\ast }b{r}_{s{\theta }_{i}}]+\{{|a{r}_{p}^{^{\prime} }|}^{2}+{|b{r}_{s}^{^{\prime} }|}^{2}\\ \quad \,\,\,\,\,\,+2\sigma \text{Im}[{(a{r}_{p}^{^{\prime} })}^{\ast }b{r}_{s}^{^{\prime} }]+[{|a|}^{2}+{|b|}^{2}+2\sigma \,{\rm{Im}}(a{b}^{\ast })]{|M|}^{2}\}/{k}_{i}^{2}{w}_{0}^{2}\}.\end{matrix}$$

We find from Eqs (2) and (3) that the displacements and energies of reflected beam contain both spin dependent and spin independent terms (terms with and without *σ*). The spin independent terms are the weighted sums of displacements of *x*_*r*_ and *y*_r_ field components, thus originating from the Goos-Hänsen (GH) effect^18^. The spin dependent terms, however, are caused by the interactions between zeroth- and first-order derivatives of the reflection coefficients of orthogonal linear polarizations. These terms vanish if the incident beam carries only one linear polarization component. Therefore, the spin dependent displacements (SDDs) in Eq. (2) are different from the displacements caused by the GH effect and the spin Hall effect of light^1, 18^. In the following, we will focus our attention on the SDDs for the cases of incident angle below and above the critical angle for total reflection.

## Results and Discussions

### Below the critical angle

For a glass prism with refractive index *n* = 1.515 (BK7 at 632.8 *nm*), the critical angle for total reflection is *θ*_*c*_ = 41.3°. When the incident angle *θ*_*i*_ is below the critical angle, i.e., *θ*_*i*_ < *θ*_*c*_, partial reflection occurs. For a linearly polarized incident beam, the spin independent terms in Eq. (2) vanish; and the RCP and LCP components of the reflected beam undergo equal displacements toward the ±*x*_*r*_ directions, which are governed by Eq. (5) in the Methods section. The SDDs Δ_±_ have complex dependences on the incident angle *θ*_*i*_ and the initial amplitude ratio between the *x*_*i*_ and *y*_*i*_ polarization components, *t* = tan*ϕ*. For each incident angle, the displacements of both RCP and LCP components, Δ_±_, vary with *t*, and there are two peak values ${{\rm{\Delta }}}_{\pm |pk}^{\pm }$ for Δ_±_ among all the *t*. The up-script “±” in ${{\rm{\Delta }}}_{\pm |pk}^{\pm }$ stands for the displacement peaks located in positive and negative *t* regions. ${t}_{pk}^{\pm }$ are positions of the displacement peaks of SDDs ${{\rm{\Delta }}}_{\pm |pk}^{\pm }$, as detailed in Eqs (7) and (8) in the Methods section.

**Figure 2:**
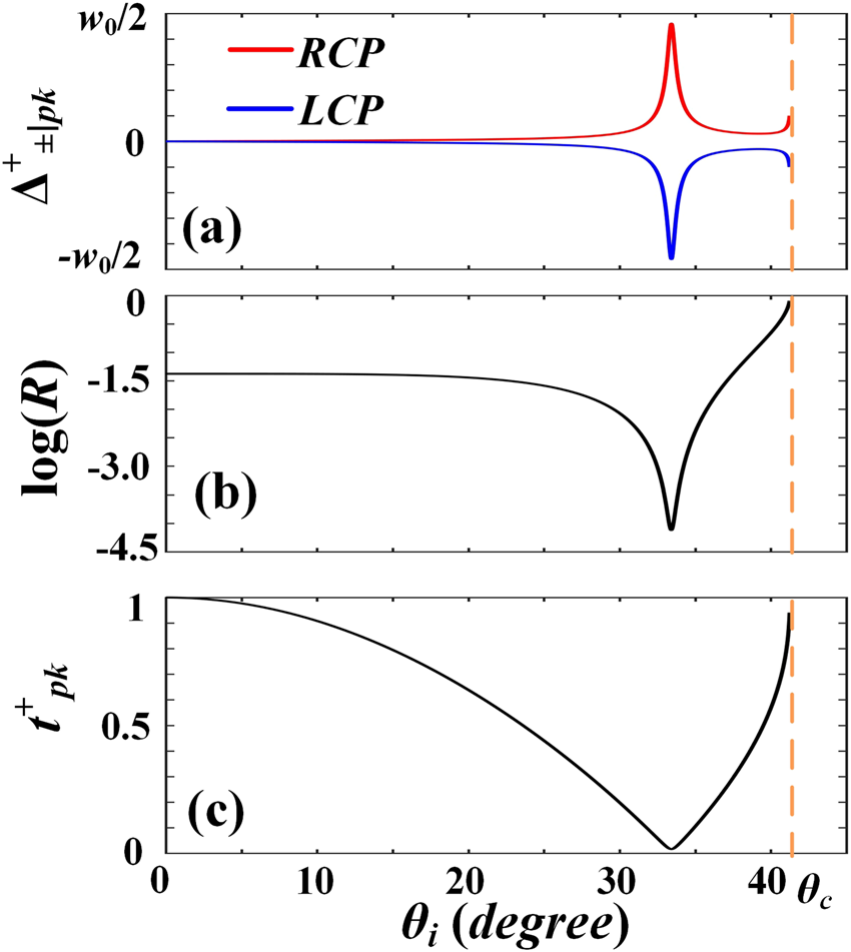
The changes of the peak displacements ${{\rm{\Delta }}}_{\pm |pk}^{+}({\theta }_{i})$ (**a**), the corresponding energy reflectivity log[*R*(*θ*_*i*_)] (**b**), and the required initial amplitude ratio ${t}_{pk}^{+}({\theta }_{i})$ (**c**) with the incident angle *θ*_*i*_. In our calculations, *w*_0_ = 25*λ*, *n* = 1.515.

Figure 2 shows respectively the dependences of the peak displacements ${{\rm{\Delta }}}_{\pm |pk}^{+}$ (a), the corresponding energy reflectivity [*R* = *W*_+_ + *W*_−_] (b), and the required initial amplitude ratio ${t}_{pk}^{+}$ (c) on the incident angle *θ*_*i*_, respectively. In our calculations, the incident beam waist is *w*_0_ = 25*λ* [*λ* being the wavelength in free space]. In general, displacement peaks $|{{\rm{\Delta }}}_{\pm |pk}^{+}|$ are smaller than *λ*. However, they are enhanced when the incident angle is near the Brewster or critical angle. At Brewster incidence *θ*_*i*_ = *θ*_*B*_, the largest $|{{\rm{\Delta }}}_{\pm |pk}^{+}|$ = 11.52*λ* is achieved, which is slightly smaller than *w*_0_/2. The initial amplitude ratio is ${t}_{pk}^{+}=0.016$ when *θ*_*i*_ = *θ*_*B*_. This suggests that a small angle between the incident linear polarization and the *x*_*i*_ axis is required to obtain the maximum spin splitting. Figure 2(b) shows that the smallest energy reflectivity, down to 7 × 10^−5^, is obtained at *θ*_*i*_ = *θ*_*B*_. Therefore, although the reflected beam undergoes the largest spin splitting at Brewster incidence, it suffers from the lowest energy reflectivity. However, the low energy reflectivity has not prevented experimental measurement of the large spin splitting around the Brewster angle^12, 16, 17^.

When the incident angle is below but close to the critical angle *θ*_*c*_, both the reflection coefficients *r*_*p*_ and *r*_*s*_ increase rapidly with incident angle *θ*_*i*_, however, at different speeds in *r*_*p*_′ and *r*_*s*_′. Therefore, according to Eq. (7), the peak displacements |${{\rm{\Delta }}}_{\pm |p{\rm{k}}}^{+}$| will increase with *θ*_*i*_ and can take large values, as shown in Fig. 2(a). For example, ${{\rm{\Delta }}}_{\pm |p{\rm{k}}}^{+}$ = ±2.7*λ* is achieved when *θ*_*i*_ = 41.25°. The pattern of peak displacement changes is somewhat similar to that of the displacement caused by the GH effect with incident angles above but near the critical angle^18^. Similarly, the energy reflectivity increases with *θ*_*i*_, and reaches 0.85 when *θ*_*i*_ = 41.25°. However, for a given beam waist *w*_0_, the incident angle *θ*_*i*_ cannot be too close to *θ*_*C*_, since the relationship Δ*θ* = *θ*_*c*_ − *θ*_*i*_ ≫ 1/*k*_*i*_ *w*_0_ must be satisfied^19^. Therefore, a larger beam waist results in an incident angle closer to the critical angle, and therefore a higher energy reflectivity and larger reachable peak displacements.

One finds from Eq. (7) and Fig. 2(a) that the maximum SDD for a given incident beam waist is obtained at the Brewster angle:

4 $${{\rm{\Delta }}}_{m}=\frac{{w}_{0}}{2\sqrt{1+{[\frac{{r}_{s{\theta }_{B}}\cot {\theta }_{B}}{{r}_{p}^{^{\prime} }}]}^{2}}}.$$

**Figure 3:**
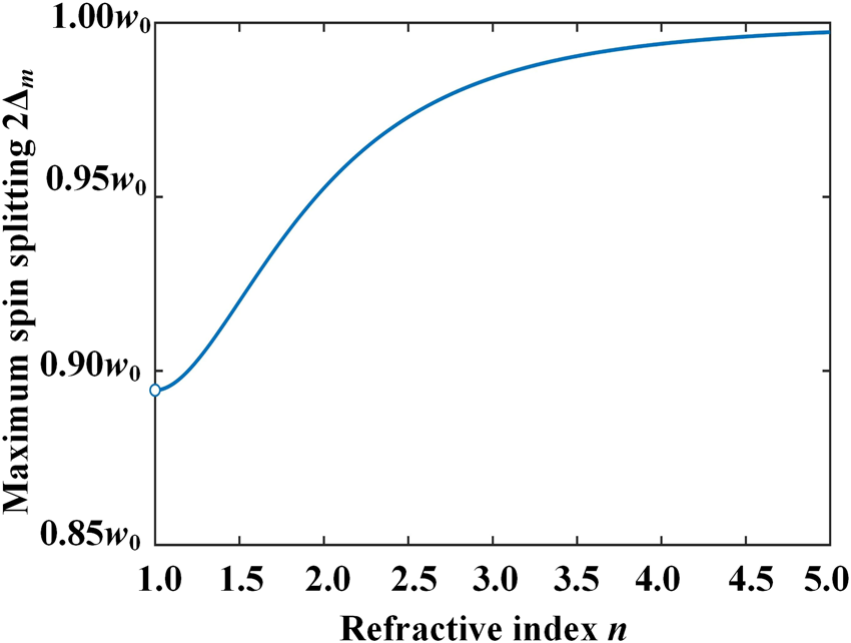
The dependence of the maximum IPSS 2Δ_*m*_ on the refractive index of the prism *n*.

At this angle, ${r}_{s{\theta }_{B}}=\,\cos \,2{\theta }_{B}=({n}^{2}-1)/({n}^{2}+1)$, ${r}_{p}^{^{\prime} }=({n}^{4}-1)/2n$. Thus, ${[{r}_{s{\theta }_{B}}\cot {\theta }_{B}/{r}_{p}^{^{\prime} }]}^{2}\,=$ ${[2n/({n}^{2}+1)]}^{4}/4.$ Therefore, the maximum IPSS, 2Δ_*m*_, grows gradually with the refractive index of the “glass” prism *n*, as shown by Fig. 3. 2Δ_*m*_ = 0.92*w*_0_ when *n* = 1.515. It is up to 0.997*w*_0_ when *n* = 5. When *n* → *∞*, the IPSS 2Δ_*m*_ tends to its upper limit of *w*_0_. It is worth noting that the IPSS can always reach *w*_0_ for 1D Gaussian incident beam because the term ${[{r}_{s{\theta }_{B}}\cot {\theta }_{B}/{r}_{p}^{^{\prime} }]}^{2}$ comes from wave-vector spreading of incident beam along *y*_*i*_ direction, which disappears for a 1D Gaussian beam. The spin splitting at *n* = 1 is not defined since there is no interface at all. However, when *n* → 1 (but not equal to 1), the maximum IPSS does not trend to zero, because the Fresnel reflection coefficients for the *s* and *p* waves (*r*_*p*_ and *r*_*s*_) as well as their derivatives (*r*_*p*_′ and *r*_*s*_′) are different, which modulate the field distributions of the two opposite spin components of reflected beam.

**Figure 4:**
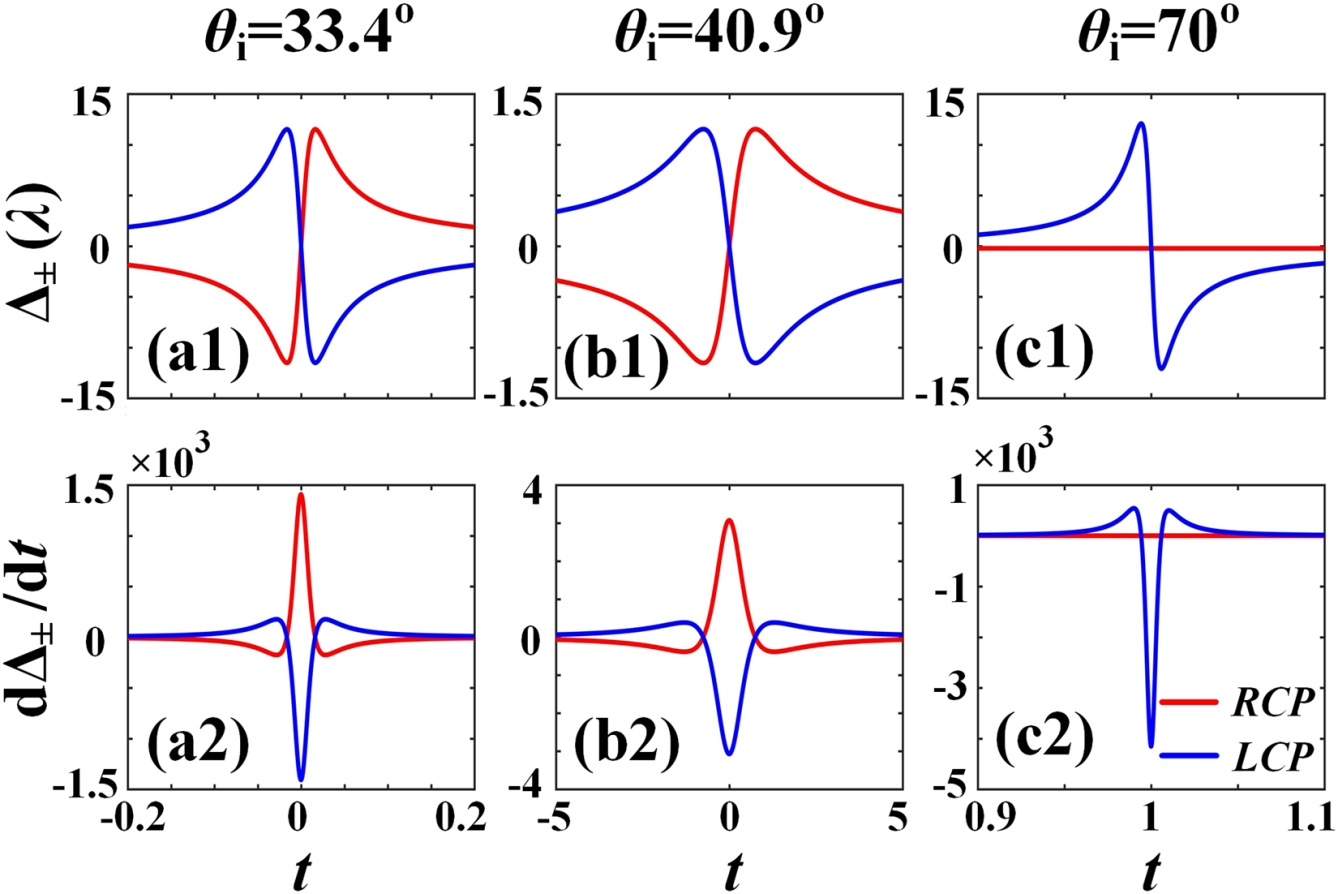
The dependences of displacements Δ_±_ and sensitivities dΔ_±_/d*t* of the RCP and LCP components of the reflected beam on the initial amplitude ratio *t* for different incident angles. (a1,a2) *θ*_*i*_ = 33.4°, (b1,b2) 40.9°, and (c1,c2) 70°.

Displacements of the RCP and LCP components of reflected beam Δ_±_ can be controlled by tuning the incident linear polarization state. As shown in Fig. 4(a1) and (b1), the SDDs Δ_±_ change signs as the initial amplitude ratio *t* crosses the zero point where Δ_±_ = 0. Δ_±_ reaches their peaks when *t* = ${t}_{pk}^{\pm }$. ${t}_{pk}^{\pm }$ = ±0.016 for Brewster incidence *θ*_*i*_ = 33.4°, and ${t}_{pk}^{\pm }$ = ±0.76 when *θ*_*i*_ = 40.9°. Considering the fact that the SDDs can be very sensitive to the initial amplitude ratio *t*, a new parameter dΔ_*σ*_/d*t* is introduced to compare this sensitivity of displacement. Figure 4(a2) and (b2) show that the peaks of dΔ_±_/d*t* occur at *t* = 0. The peaks of the RCP and LCP field components are about ±3 when *θ*_*i*_ = 40.9°. However, they are up to ±1.4 × 10^3^ when *θ*_*i*_ = 33.4°. Therefore, at Brewster incidence, a small rotation of linear polarization state will lead to dramatic changes in the SDDs of the reflected beam. The sensitivity of SDDs at Brewster incidence can be further enhanced by enlarging the incident beam waist. This feature can be fully utilized in optical sensors as previously suggested^20–22^. However, high sensitivity may cause troubles in some cases because the displacements may be easily affected by both the environment and the quality of the optical elements (polarizer in particular).

The IPSS of a Gaussian beam reflected from an interface between two dielectric media can be controlled by the incident linear polarization state. The IPSS can approach closely to its upper limit *w*_0_ for an arbitrary incident beam waist. It has been already demonstrated that, the OPSS of reflected beam at a dielectric interface reaches its maximum value when a horizontal polarized Gaussian beam is incident near the Brewster angle^12, 16^. As shown in ref. 16, the maximum spin splitting is 0.4*w*_0_ at an air-glass interface. Actually, it is smaller than 0.45*w*_0_ for all of the dielectric interfaces.

If the incident beam is a paraxial vortex one, the displacements of the RCP and LCP components of the reflected beam along the *x*_*r*_ direction [Eq. (2)] will contain two additional terms resulting respectively from the vortex-induced spatial GH effect and the coupling between the spin dependent out-of-plane angular shifts and the complex vortex structure^1, 23^. They are both linearly proportional to the vortex charge^23, 24^. The vortex-induced spatial GH shift is spin independent, and thus moves the centroids of the RCP and LCP field components together while not changing the value of the IPSS^23^. The additional spin dependent term, however, only exists for total internal reflection, since the spin dependent out-of-plane angular shift vanishes when the Fresnel coefficients for the *s* and *p* waves are both real^25^. Therefore, this additional term will influence the asymmetric spin splitting at above-critical angle incidence.

### Above the critical angle

When the incident angle is larger than the critical angle, total reflection occurs. The displacements of the RCP and LCP components of the reflected beam rely highly on the sum of the phase differences between the *x*_*i*_ and *y*_*i*_ linear polarization components, *δ*, and the phase difference between reflection coefficients, *δ*_*s*_ − *δ*_*p*_, namely, Δ*δ* = *δ* + *δ*_*s*_ − *δ*_*p*_, as shown by Eq. (9) in the Methods section. When Δ*δ* = 0, the displacements of RCP and LCP components are independent of spin. When Δ*δ* = *π*/2, both the displacements and the energies of reflected beam are spin dependent.

**Figure 5:**
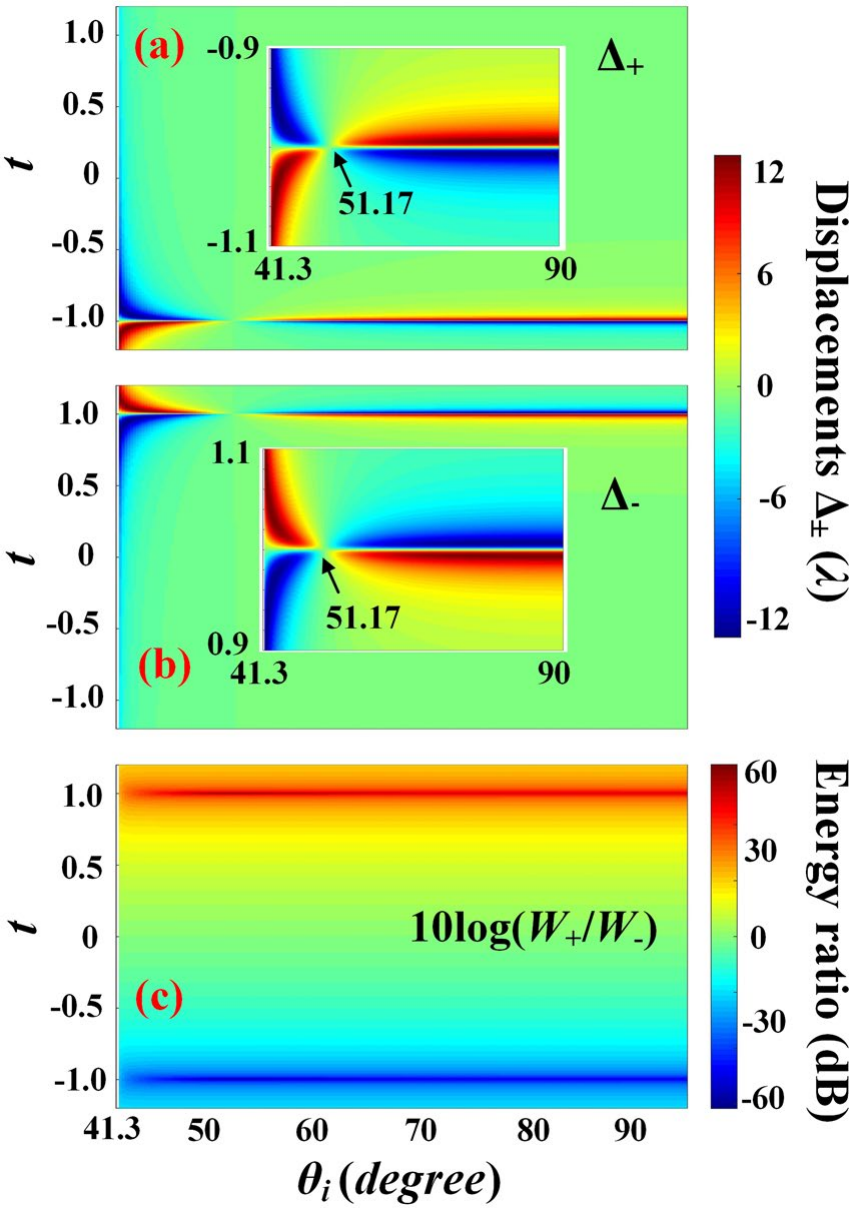
The changes of the SDDs Δ_+_ (**a**) and Δ_−_ (**b**) as functions of the incident angle *θ*_*i*_ and the initial amplitude ratio *t*, when Δ*δ* = *π*/2. (**c**) The corresponding energy ratio between the RCP and LCP components 10 log(*W*_+_/*W*_−_). Insets in (**a**) and (**b**) zoom in the ranges of *t* between *−*1.1 and −0.9 and 0.9 and 1.1, respectively.

When *σt* is away from −1, the displacements of the RCP and LCP components of the reflected beam in Eq. (11) in the Methods section can be simplified into Δ_*σ*_ = (*δ*_*p*_′ + *tδ*_*s*_′)/(1 + *σt*)/*k*_*i*_, which are the weighted sum of the *δ*_*p*_′ and *δ*_*s*_′ related displacements. Specifically, when *t* = 0 and *t* → ±∞, Δ_±_ = *δ*_*p*_′/*k*_*i*_ and *δ*_*s*_′/*k*_*i*_, respectively. In general, both the energy and the displacement of reflected beam are spin dependent. Therefore, the two spin components are asymmetrically separated^26^. This is different from the case of *θ*_*i*_ < *θ*_*C*_, where the energies of two spin components are equal, and their displacements are equal in magnitude but opposite in sign. As shown in Fig. 5(a) and (b), Δ_±_ = 0, when *σt* = −1. When *σt* is near but not equal to −1, the displacements Δ_±_ become complicated, as detailed in the Methods section. For incident angle *θ*_*i*_ = 51.17°, Δ_±_ are small. However, when the incident angle is away from 51.17°, two displacement peaks ${{\rm{\Delta }}}_{\sigma |pk}^{\pm }=\pm {w}_{0}/2$ are found for each given incident angle. The peak positions for the RCP component of the reflected beam are around *t* = −1, while they are around *t* = +1 for the LCP component as detailed in the Methods section. The energy ratios between the RCP and LCP are shown in Fig. 5(c), suggesting that the spin (RCP or LCP) component with large displacement carries much lower energy, which is the same as in the case at Brewster incidence.

As shown in Fig. 5(a) and (b), the SDDs of reflected beam Δ_±_ are sensitive to the incident polarization state when *σt* is near −1. The sensitivity near the critical angle is smaller than that near *π*/2. Figure 4(c1) and (c2) give the displacements and sensitives of the RCP and LCP components for *θ*_*i*_ = 70°. They show that the displacements of the LCP component changes rapidly with parameter *t* and reaches ±12.1*λ* when *t* = 0.994 and 1.006, respectively. The sensitivity of the LCP component is dΔ_−_/d*t* = −4.1 × 10^3^ at *t* = 0, which is about three times larger than that at Brewster incidence. However, the displacement of the RCP component is constantly equal to 0.2*λ*. This displacement is nearly equal to the displacement of total reflected beam, which is determined by the GH shift.

In the case of total internal reflection, an evanescent wave will emerge at the interface on the other side of the incidence. The electric evanescent wave has three spin angular momentum components^27^. The property of the spin of evanescent wave and its impact with the asymmetric spin splitting of reflected beam are of interest and requires further investigation^8, 27, 28^.

### Intensity distribution

**Figure 6:**
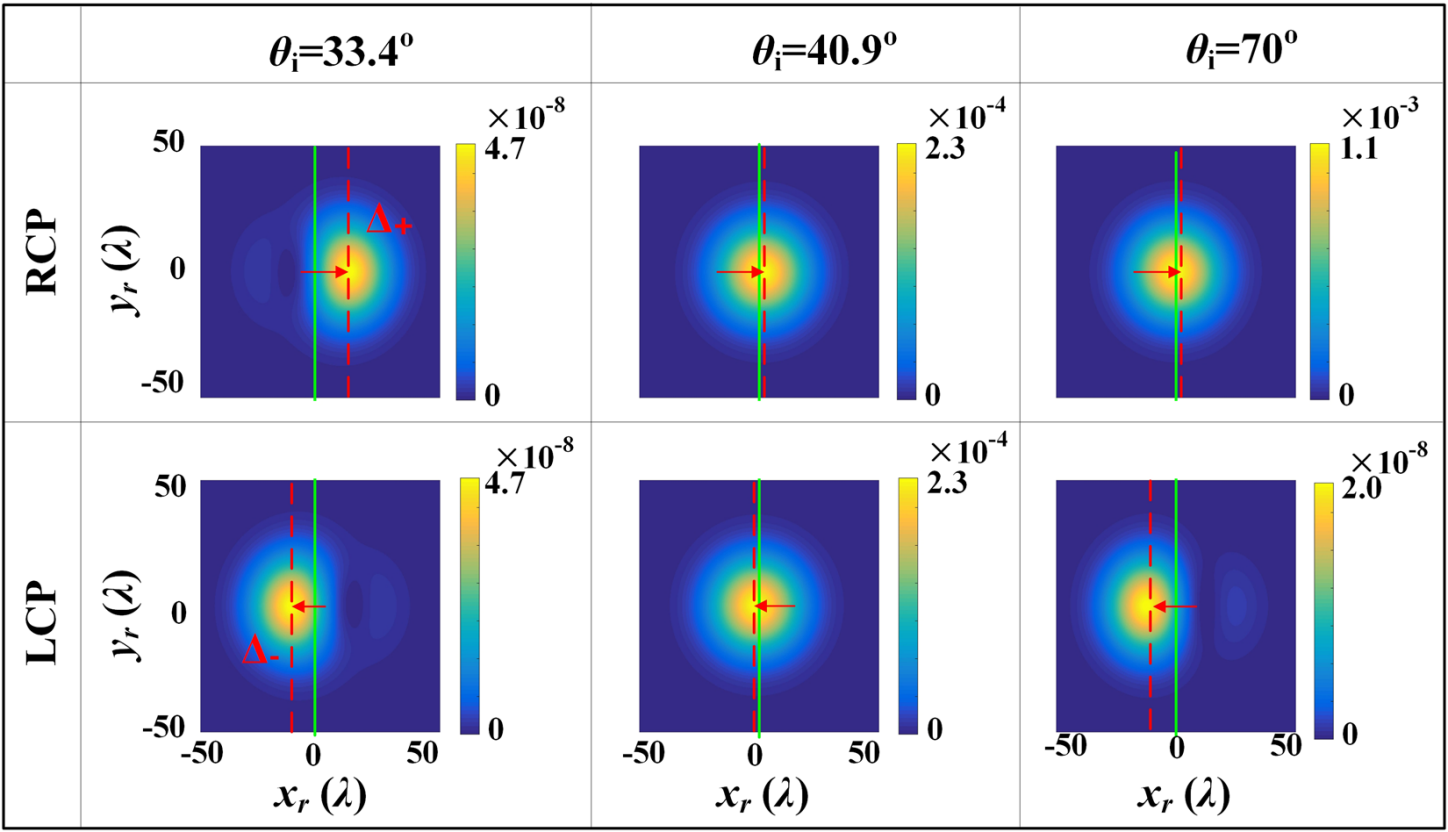
The intensity distributions of the RCP and LCP components of the reflected beam with optimal polarizations incident for three different incident angles.

Finally, we compare the intensity distributions of the two spin components of reflected beam with the optimal polarizations incident when the incident angle is equal to the Brewster angle (*θ*_*i*_ = 33.4°), slightly smaller than critical angle (*θ*_*i*_ = 40.9°), and above critical angle (*θ*_*i*_ = 70°), respectively. We calculate the intensity distributions of the RCP and LCP field components according to Eq. (1) and show the numerical results in Fig. 6. For all incident angles, the intensity distributions along the *y*_*r*_ axis are still Gaussian. However, those along the *x*_*r*_ axis change dramatically. At Brewster incidence, the intensity profiles of the RCP and LCP components shift toward ±*x*_*r*_ directions with displacements of ±11.52*λ*, and are thus strongly distorted. At *y*_*r*_ = 0, the electric field of the reflected beam is approximately ${{\bf{E}}}_{{\rm{r}}}\propto \exp [\,-{x}_{r}^{2}/{w}_{0}^{2}]\,[a{x}_{r}{r}_{p}^{^{\prime} }/{z}_{0}{\hat{{\bf{e}}}}_{rx}+ib{r}_{s{\theta }_{i}}{\hat{{\bf{e}}}}_{ry}]$. The *y*_*r*_ field component has the same Gaussian profile as the incident beam, while the *x*_*r*_ field component changes into a first order derivative of the Gaussian profile. Since there is a *π*/2 phase difference between the *x*_*r*_ and *y*_*r*_ components, the light field in the ±*x*_*r*_ domains are right- and left-handed elliptically polarized. The LCP and RCP components are mainly distributed in ±*x*_*r*_ domains. Therefore, we conclude that, the in-plane spin splitting results directly from the interaction between zeroth- and first-order derivatives of the Gaussian profile of orthogonal polarizations, while the first-order derivative of the Gaussian profile originates from the in-plane linear momentum gradients of the reflection coefficients. When *θ*_*i*_ = 40.9°, the SDDs are small, and the intensity profiles keep their initial Gaussian shape. The third column in Fig. 6 shows that the reflected beam undergoes asymmetric spin splitting when *θ*_*i*_ = 70°. The displacements of its RCP and LCP components are 0.2*λ* and −12.1*λ* respectively. The peak intensity of the LCP component is about 1 × 10^4^ times smaller than that of the RCP component. It is worth noting that, for cases of *θ*_*i*_ = 70° and *θ*_*i*_ = 33.4°, the LCP components are similar in both displacement and intensity profile.

## Conclusions

We have shown theoretically that a beam waist of *w*_0_ is the upper limit of the IPSS for a Gaussian beam reflected from a glass-air interface. For a BK7 glass prism, the spin splitting can reach 0.92*w*_0_ when the Gaussian beam incidence is exactly at the Brewster angle *θ*_*B*_. When the incident angle *θ*_*i*_ is slightly below the critical angle *θ*_*C*_, the spin splitting (displacement) can be regarded as an analogue of the GH shift slightly above the critical angle. Therefore, large splitting and high reflectivity can be simultaneously achieved when *θ*_*i*_ approaches *θ*_*C*_. For *θ*_*i*_ > *θ*_*C*_ and for a phase difference of Δ*δ* = *π*/2, one spin component of the reflected beam undergoes a relatively small displacement, while the other can undergo a large displacement of up to *w*_0_/2. Furthermore, we found that this large displacement is extremely sensitive to the incident polarization state. These findings may serve as a good foundation for further research on optical spin splitting and are useful in the development of nanophotonic devices and optical sensors.

## Methods

### The SDDs at below-critical incidence

When the incident angle of a Gaussian beam is smaller than the critical angle for total reflection, both the reflection coefficients *r*_*p*_ and *r*_*s*_ are real, and so are *r*_*p*_′ and *r*_*s*_′. Assuming that the incident beam is linearly polarized, *δ* = 0, parameters *a* and *b* are real numbers. Thus, all the terms containing imaginary functions in Eqs (2) and (3) therefore vanish; and Eq. (2) can be reduced to

5 $${{\rm{\Delta }}}_{\sigma }({\theta }_{i},t)=\frac{\sigma t[{r}_{s{\theta }_{i}}{r}_{p}^{^{\prime} }-{r}_{p{\theta }_{i}}{r}_{s}^{^{\prime} }]}{2{k}_{i}{W}_{\sigma }},$$

where

6 $${W}_{\sigma }({\theta }_{i},t)=\frac{{r}_{p{\theta }_{i}}^{2}+{t}^{2}{r}_{s{\theta }_{i}}^{2}}{2}+\frac{{({r}_{p}^{^{\prime} })}^{2}+{(t{r}_{s}^{^{\prime} })}^{2}+(1+{t}^{2}){|M|}^{2}}{2{k}_{i}^{2}{w}_{0}^{2}},$$

with *t* = tan*ϕ*. From Eqs (5) and (6) one finds that, the displacements Δ_±_ contain only spin dependent terms; and the energies carried by two opposite spin components are equal. In Eq. (6), the first two terms are related to the central wave-vector of the incident Gaussian beam. The third and fourth terms come from the wave-vector spreading of the incident beam along the *x*_*i*_ direction, while the fifth term originates from the presence of the *y*_*i*_ component of wave-vector. The SDDs Δ_±_ vary with the incident polarization state. For a given incident angle, there are two peak values for Δ_±_ among all initial amplitude ratio *t*. Therefore, the two peaks of SDDs are dependent on the incident angle and are governed by

7 $${{\rm{\Delta }}}_{\sigma |pk}^{\pm }({\theta }_{i})=\frac{\pm \sigma [{r}_{s{\theta }_{i}}{r}_{p}^{^{\prime} }-{r}_{p{\theta }_{i}}{r}_{s}^{^{\prime} }]}{2{k}_{i}\sqrt{[{r}_{p{\theta }_{i}}^{2}+(\frac{{({r}_{p}^{^{\prime} })}^{2}+{|M|}^{2}}{{k}_{i}^{2}{w}_{0}^{2}})]\,[{r}_{s{\theta }_{i}}^{2}+(\frac{{({r}_{s}^{^{\prime} })}^{2}+{|M|}^{2}}{{k}_{i}^{2}{w}_{0}^{2}})]}}.$$

The two peaks of SDDs are obtained respectively in the positive and negative *t* regions:

8 $${t}_{pk}^{\pm }({\theta }_{i})=\pm \frac{\sqrt{{r}_{p{\theta }_{i}}^{2}+(\frac{{({r}_{p}^{{}^{^{\prime} }})}^{2}+{|M|}^{2}}{{k}_{i}^{2}{w}_{0}^{2}})}}{\sqrt{{r}_{s{\theta }_{i}}^{2}+(\frac{{({r}_{s}^{^{\prime} })}^{2}+|M{|}^{2}}{{k}_{i}^{2}{w}_{0}^{2}})}}.$$

When the displacement peak of the RCP field component is positive/negative, the LCP component is at a negative/positive peak accordingly, as shown clearly in Fig. 4(a1) and (b1). More specifically, at Brewster incidence where the reflection coefficient *r*_*p*_ = 0, ${{\rm{\Delta }}}_{\sigma |pk}^{\pm }\approx \pm \sigma {{\rm{\Delta }}}_{m}$ where Δ_*m*_ is the maximum SDD for 2D Gaussian beam.

### The SDDs at above-critical incidence

When the incident angle is larger than the critical angle, the reflection coefficients are complex: *r*_*p*_ = exp(*iδ*_*p*_) and *r*_*s*_ = exp(*iδ*_*s*_). Therefore, *r*_*p*_′ = *iδ*_*p*_′exp(*iδ*_*p*_) and *r*_*s*_′ = *iδ*_*s*_′ exp (*iδ*_*s*_), where *δ*_*p*_′ and *δ*_*s*_′ are the first derivatives of *δ*_*p*_ and *δ*_*s*_, respectively. The displacements and energies of two opposite spin components of reflected beam are reduced respectively into

9 $${{\rm{\Delta }}}_{\sigma }({\theta }_{i},t)=\frac{{\delta }_{p}^{^{\prime} }+{t}^{2}{\delta }_{s}^{^{\prime} }+\sigma t[{\delta }_{p}^{^{\prime} }+{\delta }_{s}^{^{\prime} }]\,\sin \,{\rm{\Delta }}\delta }{2{k}_{i}{W}_{\sigma }},$$

10 $${W}_{\sigma }({\theta }_{i},t)=\frac{[1+{t}^{2}+2\sigma t\,\sin \,{\rm{\Delta }}\delta ]}{2}+\frac{{({\delta }_{p}^{^{\prime} })}^{2}+{t}^{2}{({\delta }_{s}^{^{\prime} })}^{2}+2\sigma t{\delta }_{p}^{^{\prime} }{\delta }_{s}^{^{\prime} }\,\sin \,{\rm{\Delta }}\delta +[(1+{t}^{2})+2t\,\sin \,\delta ]{|M|}^{2}}{2{k}_{i}^{2}{w}_{0}^{2}}.$$

where Δ*δ* = *δ* + *δ*_*s*_ − *δ*_*p*_. The variables *σ* and Δ*δ* appear in Eqs (9) and (10) always in form of *σ*sinΔ*δ*, thus the displacements and energies of reflected beam are spin independent when Δ*δ* = 0. When Δ*δ* = *π*/2, Eq. (9) can be further reduced into

11 $${{\rm{\Delta }}}_{\sigma }({\theta }_{i},t)=\frac{1}{{k}_{i}}\frac{(1+\sigma t)({\delta }_{p}^{^{\prime} }+\sigma t{\delta }_{s}^{^{\prime} })}{{(1+\sigma t)}^{2}+\frac{{[{\delta }_{p}^{^{\prime} }+\sigma t{\delta }_{s}^{^{\prime} }]}^{2}}{{k}_{i}^{2}{w}_{0}^{2}}+\frac{[(1+{t}^{2})+2t\,\sin \,\delta ]|M{|}^{2}}{{k}_{i}^{2}{w}_{0}^{2}}}.$$

When *σt* = −1, Δ_±_ = 0. When *σt* is near but not equal to −1, the first term in the denominator of Eq. (11) is very small, thus the last two terms of denominator cannot be neglected. For incident angle *θ*_*i*_ = 51.17°, *δ*_*p*_′ = *δ*_*s*_′, the third terms in denominator is larger than the first two terms when *σt* is near -1. The SDDs Δ_±_ therefore almost vanish. However, when the incident angle is away from 51.17°, the second term of the denominator in Eq. (11) is much larger than the third term. Therefore, the third term can be neglected. In this situation, given an incident angle, two peaks ${{\rm{\Delta }}}_{\sigma |pk}^{\pm }({\theta }_{i})=\pm {w}_{0}/2$ can be found at ${t}_{pk}^{\pm }({\theta }_{i})=\sigma ({{\delta }_{p}}^{^{\prime} }\pm {k}_{i}{w}_{0})/({{\delta }_{s}}^{^{\prime} }\pm {k}_{i}{w}_{0})$, as shown in Fig. 5(a) and (b).
